# Time of Detection of Liver Metastases and Survival in Patients With Colorectal Cancer

**DOI:** 10.1001/jamanetworkopen.2026.2088

**Published:** 2026-03-17

**Authors:** Ruby Kemna, Hannah H. Schulz, Mahsoem Ali, Madelon Dijkstra, Susan van der Lei, Marinde J. G. Bond, Cornelis J. A. Punt, Inez M. Verpalen, Tineke E. Buffart, Rutger-Jan Swijnenburg, Geert Kazemier, Martijn R. Meijerink

**Affiliations:** 1 Amsterdam University Medical Center, Department of Surgery, VU University, Amsterdam, the Netherlands; 2 Cancer Center Amsterdam, the Netherlands; 3 Amsterdam UMC, Department of Radiology and Nuclear Medicine, VU University, Amsterdam, the Netherlands; 4 University Medical Centre Utrecht, Department of Epidemiology, Julius Centre for Health Sciences and Primary Care, Utrecht University, Utrecht, the Netherlands; 5 Amsterdam UMC, Department of Medical Oncology, VU University, Amsterdam, the Netherlands

## Abstract

**Importance:**

Colorectal cancer liver metastases (CRLM) are a common manifestation of colorectal cancer, and clinical decision-making for these patients is complex. The timing of CRLM detection, defined as synchronous, early metachronous, or late metachronous, is hypothesized to influence survival outcomes, but the prognostic implications of these classifications remain unclear.

**Objective:**

To determine whether the timing of CRLM detection is associated with overall survival (OS) in patients with up-front locally treatable or initially nonlocally treatable CRLM.

**Design, Setting, and Participants:**

Multicenter cohort study including adult patients from the Amsterdam Colorectal Liver Met Registry and the phase 3 CAIRO5 trial between 2000 and 2024, with analyses conducted from March 2025 to January 2026. Patients were followed up for a median (IQR) of 44 (41-48) months. Survival outcomes were analyzed using multivariable Cox regression models. Inclusion criteria were up-front locally treatable or initially nonlocally treatable CRLM, with key clinical and demographic characteristics recorded.

**Exposure:**

Timing of CRLM detection categorized as synchronous (detected before or at primary CRC diagnosis or during surgery), early metachronous (within 12 months of diagnosis or surgery), or late metachronous (more than 12 months after surgery), according to European-African Hepato-Pancreato-Biliary Association consensus guidelines.

**Main Outcomes and Measures:**

OS from diagnosis of CRLM, stratified by CRLM timing and treatment received. Multivariable analysis accounted for tumor number, size, variant status, carcinoembryonic antigen levels, and treatment strategy.

**Results:**

A total of 1250 patients were included (median [IQR] age, 63 [56-72] years; 823 [65.9%] men), of whom 817 (65%) had synchronous CRLM, 208 (17%) early metachronous CRLM, and 225 (18%) late metachronous CRLM. Among all patients, 676 (54%) died during follow-up. Synchronous CRLM were associated with worse OS compared with early metachronous CRLM (hazard ratio [HR], 0.79; 95% CI, 0.64-0.97) and late metachronous CRLM (HR, 0.54; 95% CI, 0.43-0.68). After adjustment for tumor and treatment factors, timing of detection of CRLM was not independently associated with OS.

**Conclusions and Relevance:**

In this retrospective multicenter cohort study, timing of CRLM detection was not associated with OS when accounting for tumor characteristics and treatment strategy. These findings suggest that synchronicity may primarily reflect underlying tumor biology rather than directly influencing survival, emphasizing that clinical decisions should focus on synchronicity in combination with tumor burden, variant status, and local treatment.

## Key Points

**Question:**

Is the timing of colorectal cancer liver metastasis (CRLM) detection, defined as synchronous, early metachronous, or late metachronous, associated with overall survival?

**Findings:**

In this cohort study of 1250 patients, synchronous CRLM initially appeared to be associated with worse survival; however, timing of detection was not an independent factor after adjusting for tumor number, size, variant status, carcinoembryonic antigen levels, and treatment strategy. The ability to undergo local treatment had the greatest association with improved survival.

**Meaning:**

These findings suggest synchronicity is not independently associated with a survival benefit and may instead be indicative of underlying tumor biology, with synchronous metastases occurring earlier in the disease course.

## Teaser

This cohort study evaluates whether the timing of colorectal cancer liver metastasis detection is associated with overall survival in patients with up-front locally treatable or initially nonlocally treatable colorectal cancer liver metastasis.

## Introduction

The liver is the most common site of metastases in patients with colorectal cancer (CRC), and colorectal liver metastases (CRLM) are a key determinant of prognosis.^1,2^ The onset of metastatic disease remains an important oncological criterion in the management of CRLM, as the timing of CRLM diagnosis is hypothesized to be associated with survival outcomes. CRLM are often categorized into 3 subtypes based on time interval from detection or diagnosis of the primary CRC to diagnosis of CRLM. They are classified as occurring synchronous, early metachronous, or late metachronous in relation to the primary CRC diagnosis.^3^ Several studies have shown a worse prognosis of patients with synchronous compared with metachronous CRLM, hypothesizing that synchronous CRLMs have a more aggressive tumor biology with a higher risk of disease recurrence and a lower OS compared with metachronous CRLM.^4,5,6,7^ Other studies found similar 5-year overall survival (OS) rates between these groups, implying limited prognostic value of CRLM detection time subtypes.^7,8^

The prognostic value of synchronicity should be interpreted with caution as various time intervals for the definition of synchronous CRLM are used.^3,4,5,9^ In 2015, the Expert Group on Oncosurgery Management of Liver Metastases (EGOSLIM) proposed a multidisciplinary international consensus on the management and definition of synchronous CRLM to adopt a standardized definition, allowing for consistent research reporting and more reliable conclusions.^4^ This consensus defined synchronous CRLM as liver metastases that are detected at the same time or before the diagnosis of the primary CRC; early metachronous metastases as those detected within 12 months of diagnosis or surgery of the primary CRC; and late metachronous as those detected after more than 12 months. Despite the EGOSLIM consensus, variation in defining synchronicity persisted in literature.^4,5,10^ Moreover, diagnostic methods changed over the years, and treatment pathways became more complex because of biological and technical advances.^11^ To incorporate these developments, an improved multisocietal European-African Hepato-Pancreato-Biliary Association (E-AHPBA) consensus on terminology and management of patients with synchronous CRLM was proposed in 2023.^12^ The definitions remained mostly consistent with the EGOSLIM consensus of 2015. However, the definition of synchronous CRLM was expanded to include patients with CRLM detected during surgery of the primary CRC, which were previously considered to be early metachronous CRLM.

Although the onset of metastatic disease remains an important oncological criterion in the management of CRLM and consensus on synchronicity definitions has been achieved by the E-AHPBA, the prognostic implications of stratifying CRLM still remain unclear. Therefore, the aim of this study was to assess the value of synchronous, early metachronous, and late metachronous CRLM as defined in the E-AHPBA consensus for patients with up-front and initially not locally treatable CRLM.

## Methods

### Study Population and Data Extraction

This large cohort study included patients of 2 cohorts: (1) the Amsterdam Colorectal Liver Met Registry (AmCORE registry) and (2) the multicenter phase 3 CAIRO5 trial. The AmCORE registry is a single-center, prospective database at Amsterdam University Medical Center that includes patients treated locally for CRLM. The CAIRO5 trial is a multicenter phase 3 randomized study evaluating different systemic treatment regimens for patients with initially unresectable CRLM based on tumor characteristics. A detailed overview on both cohorts, including diagnostic work-up and follow-up are described in eAppendix 1 in Supplement 1. The medical ethics review committee of the Amsterdam University Medical Centre in Amsterdam, The Netherlands, approved this study, and due to the retrospective nature of the study, the need for informed consent was waived. This study was reported in accordance with the Strengthening the Reporting of Observational Studies in Epidemiology (STROBE) reporting guideline for cohort studies.^13^ The following patient-, disease-, and treatment-related characteristics were collected from both patient cohorts: sex, age, primary colorectal tumor location, molecular profile (rat sarcoma viral oncogene homologue [*RAS*], V-raf murine sarcoma viral oncogene homologue [*BRAF*] V600E, or mismatch repair [MMR] proficient or deficient), number of CRLM at diagnosis, largest CRLM diameter at diagnosis, carcinoembryonic antigen (CEA) level at diagnosis, Fong score, type of local treatment, and any systemic treatment before local treatment (eAppendix 2 in Supplement 1).

### Primary and Secondary Outcomes

The primary outcome was OS, and to mitigate the risk of immortal time bias, OS was defined as the time from the diagnosis of CRLM to death from any cause, and patients were right-censored at the time of last follow-up if they were still alive at the end of follow-up. Defining OS from the time of CRLM diagnosis provides a more accurate and clinically relevant estimate of prognosis across the timing categories.^14^ For illustrative purposes, OS was also calculated from the time of primary CRC diagnosis in supplementary analyses. OS was assessed across 3 subgroups. First, comparisons were made among all included patients based on the timing of CRLM diagnosis: synchronous, early, and late metachronous. Second, OS differences according to the timing of CRLM diagnosis were compared between the AmCORE and CAIRO5 study cohorts and substratified by treatment strategy.

Secondary outcomes included OS from diagnosis of primary tumor in both cohorts, and distant progression-free survival (DPFS) in the AmCORE cohort (eAppendix 3 in Supplement 1). For DPFS, death was treated as a competing risk.

### Definitions of Synchronous, Early Metachronous, and Late Metachronous CRLM

Definitions of synchronous, early, and late metachronous CRLM were used according to the E-AHPBA, European Society of Surgical Oncology, European Society of Coloproctology, European Society of Gastrointestinal and Abdominal Radiology, and Cardiovascular and Interventional Radiological Society of Europe consensus terminology.^12^ Synchronous CRLM was defined as metastases detected before or at the time of diagnosis of the primary CRC, including CRLM detected during surgery of the primary CRC. Metachronous CRLM was defined as all metastases that have been diagnosed after the initial cross-sectional imaging and surgery to detect and treat the primary CRC. A distinction is made between early metachronous CRLM, which was defined as metastases detected within 12 months after diagnosis of the primary CRC, and late metachronous CRLM, defined as metastases detected more than 12 months after the primary CRC diagnosis.

### Statistical Analysis

All baseline characteristics were compared across 3 groups: (1) patients with synchronous, early metachronous, and late metachronous CRLM; (2) AmCORE and CAIRO5 cohorts; and (3) patients who received up-front local treatment, induction systemic therapy followed by local treatment, and induction systemic therapy without subsequent local treatment. Categorical variables were presented as frequencies and percentages, and were compared across groups using the Pearson χ^2^ test. Continuous variables were reported as median (IQR), and were compared between groups using the Kruskal-Wallis test.

Unadjusted OS and DPFS were estimated using the Kaplan-Meier method, and differences were evaluated using the log-rank test and Cox proportional hazards model. Results were presented as hazard ratios (HRs) with 95% CIs. Right-censoring was applied for patients still alive at the last follow-up time point. Patients experiencing competing events were treated as censored at the time of the event.

Multivariable analysis was performed using the Cox regression model to correct for the following prespecified potential confounders: patient cohort (AmCORE *vs* CAIRO5), sex, age, primary tumor location (rectum, right-sided colon, or left-sided colon), variant status (*RAS* wildtype, variant, and unknown and *BRAF* wildtype, variant, and unknown), number of CRLM at diagnosis, largest tumor diameter at diagnosis, CEA level at diagnosis of CRLM (log-transformed for right-skewed data), Fong score, type of local treatment (resection, thermal ablation, combination of resection and thermal ablation, or no local treatment). Continuous variables other than CEA levels were modeled using restricted cubic splines with 3 knots to capture any nonlinear covariate-outcome associations. Multiplicative interaction terms between confounders were not considered in the model specification process. Confounders were selected based on domain knowledge and previous literature (ie, variable selection methods were not performed). All confounders were determined at baseline, that is, before or shortly after CRLM detection. The estimated prognostic value of all covariates included in the multivariable model was determined by calculating the likelihood ratio χ^2^ value; briefly, higher χ^2^ values indicate higher independent estimated prognostic value, irrespective of the measurement scale of the variable.

Associations between the timing of CRLM detection (synchronous, early metachronous, and late metachronous) and included parameters were tested in multivariable Cox regression models for OS, adjusted for the covariates that were included in the main multivariable Cox proportional hazards model. The proportional hazards assumptions was assessed using the Grambsch–Therneau test and visual inspection of Schoenfeld residuals. The significance of each variable was determined using a likelihood ratio test to calculate the corresponding *P* value and eventually presented as adjusted HRs (aHRs) with 95% CIs. To assess whether the association of synchronicity with OS differed between patient cohorts (CAIRO5 vs AmCORE) and between treatment strategies, the multivariable Cox regression model was extended with a synchronicity-by-cohort interaction term, and in a separate analysis, with a synchronicity-by-treatment interaction term.

All statistical analyses were performed using RStudio, version 4.5.0. (R Project for Statistical Computing,). A *P* value lower than .05 was considered significant. All figures were generated using GraphPad Prism software version 10.2.0 (GraphPad Software).

## Results

### Study Population and Baseline Characteristics

A total of 1250 patients were included in this retrospective study cohort, including 729 consecutive patients who were retrieved from the AmCORE registry and all 521 patients from the CAIRO5 trial (eFigure 1 in Supplement 1). Of all patients, 817 (65.4%) had synchronous CRLM, 208 (16.6%) had early metachronous CRLM, and 225 (18.0%) had late metachronous CRLM (Table 1). The distribution of synchronicity varied significantly between the AmCORE and CAIRO5 study cohorts (synchronous: 369 patients [50.6%] vs 448 [86.0%]; early metachronous: 165 [22.6%] vs 43 [8.3%]; late metachronous: 195 [26.8%] vs 30 [5.8%]; *P* < .001), with a higher proportion of synchronous CRLM in the CAIRO5 trial. The size of the largest CRLM was larger in patients with synchronous CRLM compared with early and late metachronous CRLM (median [IQR], 34 [21-57] mm; 26 [20-35] mm; and 25 [18-38] mm; *P* < .001). Also, CEA levels at diagnosis were higher among patients with synchronous CRLM (median [IQR], 25 [6-151], 8 [3-35], and 7 [4-15] ng/mL; *P* < .001; to convert ng/mL to μg/L, multiply by 1), compared with patients with early and late metachronous CRLM. There were numerically higher rates of *RAS* and *BRAF *V600E variants in patients with synchronous CRLM, with variant distribution significantly different between groups (synchronous: 274 of 817 patients [33.5%] and 38 of 817 patients [4.7%]; early metachronous: 35 of 208 patients [16.9% ] and 6 of 208 patients [2.9%]; late metachronous: 29 of 225 patients [12.9%] and 1 of 225 patients [0.4%] with *RAS* and *BRAF *V600E, respectively; *P* < .001 for all). However, the proportion of unknown variant status was high, particularly within the AmCORE cohort (514 of 729 patients [70.7%] and 530 of 729 patients [72.8%] with unknown *RAS* and *BRAF *V600E, respectively). Administration of induction systemic therapy followed by local treatment was most common in synchronous CRLM (synchronous: 411 of 791 patients [52.0%]; early metachronous: 71 of 194 patients [36.6%]; late metachronous: 54 of 209 patients [25.8%]; *P* < .001), while up-front local treatment was more common in metachronous disease (synchronous: 187 of 791 patients [23.6%]; early metachronous: 115 of 194 patients [59.3%]; late metachronous: 147 of 209 patients [70.3%]; *P* < .001).

**Table 1.** Patient-, Tumor- and Treatment-Related Characteristics by Colorectal Cancer Liver Metastasis (CRLM) Detection Time Interval

Characteristic	Patients, No. (%)				*P* value
	Total (N = 1250)	Synchronous (n = 817)	Early metachronous (n = 208)	Late metachronous (n = 225)	
Patient-related					
Patient cohort					
AmCORE registry	729 (58.3)	369 (45.2)	165 (79.3)	195 (86.7)	<.001
CAIRO5 trial	521 (41.7)	448 (54.8)	43 (20.7)	30 (13.3)	
Sex					
Male	823 (65.9)	521 (63.9)	146 (70.2)	156 (69.3)	.12
Female	425 (34.1)	294 (36.1)	62 (29.8)	69 (30.7)	
Age, median (IQR), y	63 (56-72)	62 (55-70)	65 (57-73)	69 (61-75)	<.001
Tumor-related					
Primary tumor location					
Rectum	455 (36.4)	284 (34.8)	89 (42.8)	82 (36.6)	.22
Right-sided colon	299 (23.9)	195 (23.9)	50 (24.0)	54 (24.1)	
Left-sided colon	495 (39.6)	338 (41.1)	69 (33.2)	88 (39.3)	
Molecular profile					
* RAS*					
Wildtype	396 (31.7)	310 (37.9)	48 (23.2)	38 (17.0)	<.001
Variant	338 (27.1)	274 (33.5)	35 (16.9)	29 (12.9)	
Unknown	514 (41.2)	233 (28.5)	124 (59.9)	157 (70.1)	
* BRAF*					
Wildtype	674 (54.0)	536 (65.6)	73 (35.1)	65 (29.0)	<.001
Variant	45 (3.6)	38 (4.7)	6 (2.9)	1 (0.4)	
Unknown	530 (42.4)	243 (29.7)	129 (62.0	158 (70.5)	
MMR					
Proficient	401 (32.1)	253 (31)	74 (35.6)	74 (33.0)	.12
Deficient	9 (0.7)	5 (0.6)	4 (1.9)	0	
Unknown	839 (67.2)	559 (68.4)	130 (62.5)	150 (67.0)	
No. of CRLM at diagnosis, median (IQR)	5 (2-11)	7 (3-15)	3 (1-6)	1 (1-3)	<.001
Largest tumor diameter at diagnosis, median (IQR), mm	30 (20-49)	34 (21-57)	26 (20-35)	25 (18-38)	<.001
CEA levels at diagnosis, median (IQR), ng/mL	15 (5-92)	25 (6-151)	8 (3-35)	7 (4-15)	<.001
Fong score					
1	132 (17.3)	39 (7.2)	27 (26.5)	66 (56.9)	.01
2	275 (36.1)	203 (37.4)	39 (38.2)	33 (28.4)	
3	230 (30.2)	185 (34.1)	29 (28.4)	16 (13.8)	
4	114 (15)	108 (19.9)	5 (4.9)	1 (0.9)	
5	10 (1.3)	8 (1.5)	2 (2.0)	0	
Treatment-related					
Local treatment					
Resection	412 (33.0)	244 (29.9)	77 (37.0)	91 (40.4)	<.001
TA	274 (21.9)	139 (17.0)	60 (28.8)	75 (33.3)	
Resection and TA	355 (28.4)	241 (29.5)	63 (30.3)	51 (22.7)	
No local treatment	209 (16.7)	193 (23.6)	8 (3.8)	8 (3.6)	
Systemic therapy (induction)					
No systemic therapy	449 (37.6)	187 (23.6)	115 (59.3)	147 (70.3)	<.001
Induction systemic therapy followed by local treatment	536 (44.9)	411 (52.0)	71 (36.6)	54 (25.8)	
Induction systemic therapy not followed by local treatment	209 (17.5)	193 (24.4)	8 (4.1)	8 (3.8)	

Abbreviations: AmCORE, Amsterdam Colorectal Liver Met Registry; CEA, carcinoembryonic antigen; MMR, DNA mismatch repair; TA, thermal ablation (including microwave ablation and radiofrequency ablation).

SI conversion factor: To convert ng/mL to μg/L, multiply by 1.

Differences in baseline characteristics between patients of the AmCORE and CAIRO5 trial cohorts and the different treatment strategy groups (up-front local treatment or after induction therapy and induction therapy without subsequent local treatment) are described in eTables 1 and 2 in Supplement 1, respectively.

### Survival Outcomes

**Figure 1.**
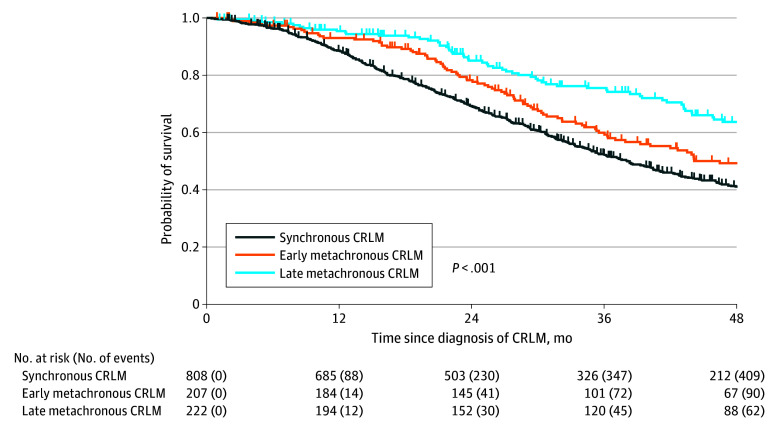
Kaplan-Meier Curve for Unadjusted Overall Survival From Diagnosis of Colorectal Cancer Liver Metastasis (CRLM) in Patients With Synchronous, Early Metachronous, and Late Metachronous CRLM Compared with log-rank test (*P* < .001).

After a median (IQR) follow-up time of 44.0 (41.0-48.0) months, 676 patients (54.1%) had died and 218 patients were lost to follow-up. Figure 1 presents the unadjusted OS Kaplan-Meier curves from diagnosis of CRLM in synchronous, early metachronous, and late metachronous CRLM. In patients with synchronous CRLM, the median OS was 38.3 (95% CI, 35.4-42.4) months with 1-, 3-, and 5-year OS rates of 88.8% (95% CI, 86.7%-91.1%), 52.6% (95% CI, 49.0%-56.4%), and 31.5% (95% CI, 27.9%-35.6%), respectively. Patients with early metachronous CRLM had 1-, 3-, and 5-year OS rates of 93.0% (95% CI, 89.6%-96.6%), 61.0% (95% CI, 54.3%-68.5%), and 39.3% (95% CI, 32.0%-48.2%), respectively. Patients with late metachronous CRLM showed a median OS of 64.3 (95% CI, 42.4-90.6) months with 1-, 3-, and 5-year OS rates of 94.3% (95% CI, 91.3%-97.5%), 76.1% (95% CI, 70.2%-82.5%), and 52.1% (95% CI, 44.5%-60.9%). Patients with synchronous CRLM showed a lower OS compared with patients with early metachronous CRLM (HR, 0.79; 95% CI, 0.64-0.97; *P* = .02), and compared with those with late metachronous CRLM (HR, 0.54; 95% CI, 0.43-0.68; *P* < .001).

**Figure 2.**
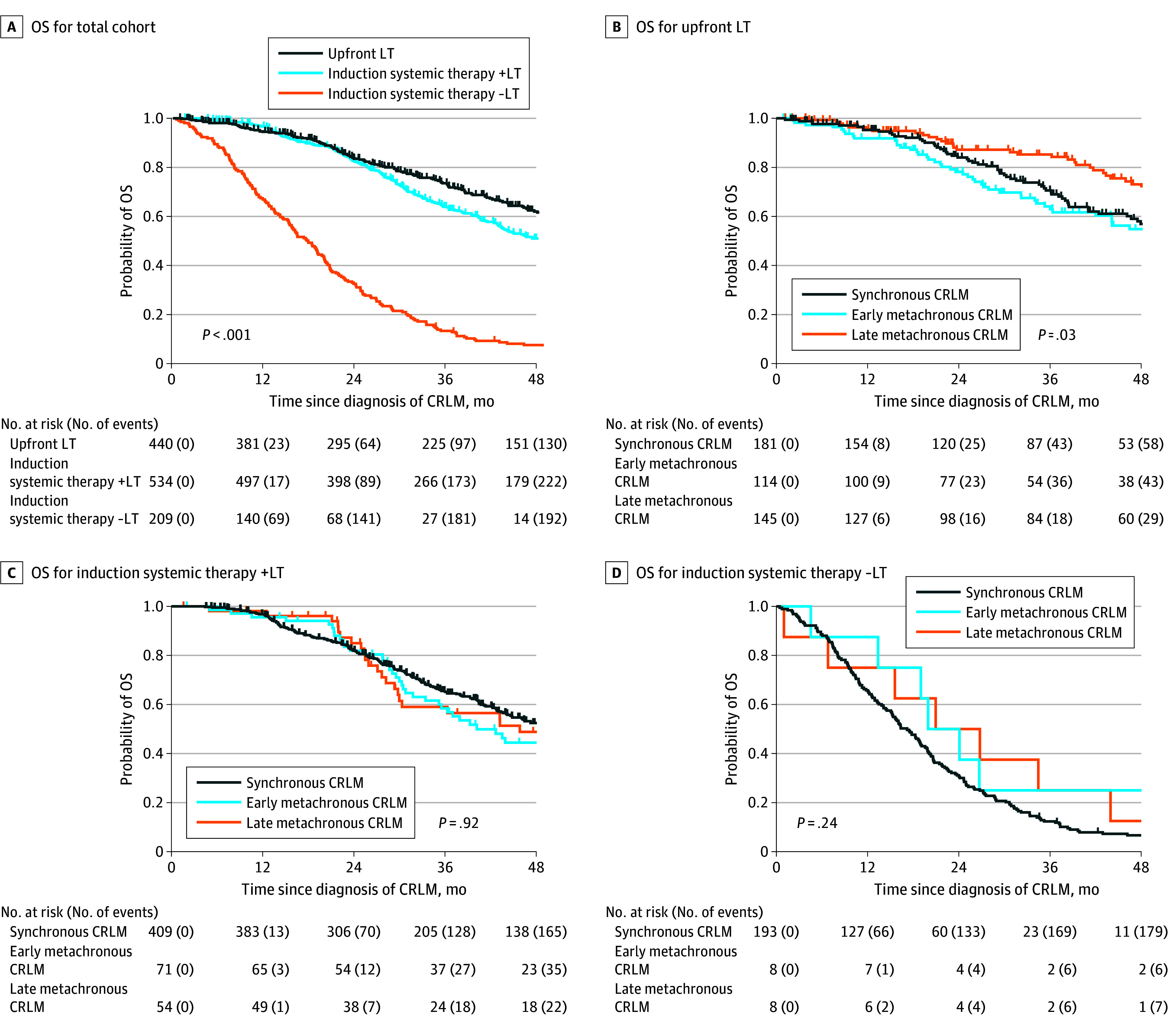
Kaplan-Meier Curves for Unadjusted Overall Survival (OS) From Diagnosis of Colorectal Cancer Liver Metastasis (CRLM) in Different Patient Subgroups LT indicates local treatment.

OS was compared across treatment strategies; compared with up-front local treatment, induction systemic therapy was associated with worse survival (HR, 1.38; 95% CI, 1.14-1.67; *P* = .001), while systemic therapy without subsequent local treatment showed a markedly higher hazard of death (HR, 6.50; 95% CI, 5.25-8.04; *P* < .001) (Figure 2A). In addition, Figure 2B through D shows that OS was similar across the synchronous, early metachronous, and late metachronous subgroups. However, in the up-front locally treated cohort (Figure 2B), patients with late metachronous CRLM showed significantly better OS compared with those with synchronous or early metachronous disease.

In supplementary analyses, OS was also markedly different between synchronous, early, and late metachronous CRLM when calculated from the date of primary tumor diagnosis (synchronous vs early metachronous: HR, 0.64; 95% CI, 0.52-0.79; *P* < .001; synchronous vs late metachronous: HR, 0.28; 95% CI, 0.22-0.36; *P* < .001) (eFigure 2 in Supplement 1). Moreover, for patients with CRLM in the AmCORE registry, patients with early metachronous CRLM showed the worst overall survival, and within the CAIRO5 trial, patients with synchronous CRLM showed the worst OS (eFigure 3 in Supplement 1).

During follow-up, 774 cases of distant progression were observed within the AmCORE cohort. Patients with synchronous CRLM had a lower DPFS rate compared with those with early metachronous CRLM (HR, 0.82; 95% CI, 0.68-0.98; *P* = .03), and compared with those with late metachronous CRLM (HR, 0.52; 95% CI, 0.43-0.63; *P* < .001) (eFigure 4 in Supplement 1).

### Variables Associated With Survival Outcomes

**Figure 3.**
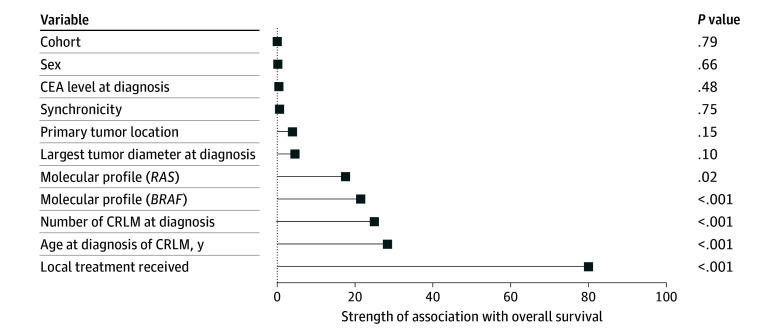
Independent Prognostic Value of Included Variables for Overall Survival Strength of association of a variable is represented on the x-axis as χ^2^ values. Higher χ^2^ values indicate stronger associations with overall survival in the multivariable analysis. *P* values and χ^2^ values were obtained from likelihood ratio tests for the full multivariable Cox regression model. CEA indicates carcinoembryonic antigen; CRLM, colorectal cancer liver metastasis.

After adjusting for all potential confounders in multivariable Cox regression analysis, the timing of CRLM detection was not independently associated with prognosis for OS, either in the overall analysis or when comparing synchronous vs early metachronous CRLM (aHR, 0.99; 95% CI, 0.75-1.31), and synchronous vs late metachronous CRLM (aHR, 0.80; 95% CI, 0.58-1.08]) (Table 2, Figure 3). Number of CRLM at diagnosis, age, and receipt of local treatment were the strongest factors associated with OS, even after adjusting for clinical and size-based variables (Figure 3). There was no evidence that the association of synchronicity with OS differed between the CAIRO5 and AmCORE cohorts or between treatment strategies.

**Table 2.** Cox Regression Models for Association Between Potential Confounders and Mortality

Variable	Total (n = 1237)	Multivariable analysis, aHR (95% CI)	*P* value
Patient cohort			
AmCORE registry	716 (57.9)	1 [Reference]	.18
CAIRO5 trial	521 (42.1)	1.02 (0.65-1.06)	
Synchronicity			
Synchronous CRLM	808 (65.3)	1 [Reference]	.31
Early metachronous CRLM	207 (16.7)	0.99 (0.75-1.31)	
Late metachronous CRLM	222 (17.9)	0.80 (0.58-1.08)	
Sex			
Male	815 (66)	1 [Reference]	.79
Female	420 (34)	0.98 (0.81-1.17)	
Age (per 10 y increase)	1233 (99.7)	1.23 (1.11-1.35)	<.001
Primary tumor location			
Rectum	452 (36.6)	1 [Reference]	.19
Left-sided colon	489 (39.6)	0.91 (0.74-1.12)	
Right-sided colon	295 (23.9)	1.13 (0.91-1.42)	
Molecular profile *RAS*			
Unknown	503 (40.7)	1 [Reference]	.001
Wildtype	394 (31.9)	2.14 (0.82-5.60)	
Variant	338 (27.4)	2.96 (1.15-7.58)	
Molecular profile *BRAF*			
Unknown	519 (42.0)	1 [Reference]	<.001
Wildtype	672 (54.4)	0.58 (0.22-1.49)	
Variant	45 (3.6)	1.38 (0.49-3.82)	
Number CRLM at diagnosis (per 5 lesions increase)	1202 (97.1)	1.06 (1.04-1.08	<.001
Largest tumor diameter at diagnosis (mm) (per 20mm increase)	1150 (93.0)	1.03 (1.02-1.03)	.43
CEA level at diagnosis (per 50 ng/mL increase)	953 (77.0)	1.00 (1.00-1.00)	.78
Treatment			
Resection	402 (32.5)	1 [Reference]	<.001
TA	273 (22.1)	1.08 (0.78-1.50)	
Resection and TA	353 (28.5)	1.05 (0.82-1.35)	
No local treatment (after induction therapy)	209 (16.9)	3.12 (2.40-4.05)	

Abbreviations: aHR, adjusted hazard ratio; AmCORE, Amsterdam Colorectal Liver Met Registry; CEA, carcinoembryonic antigen; CRLM, colorectal cancer liver metastasis; TA, thermal ablation (including microwave ablation and radiofrequency ablation).

SI conversion factor: To convert ng/mL to μg/L, multiply by 1.

## Discussion

This international cohort study showed that the timing of CRLM detection, based on the recent E-AHPBA consensus, was not significantly associated with OS. Rather, the strongest factor associated with improved survival was the ability to undergo local treatment. In other words, patients with synchronous metastases had comparable OS with those with metachronous metastases when survival was calculated from the time of CRLM diagnosis. At the same time, CIs were generally wide and were compatible with a substantial reduction in mortality risk with late metachronous disease or a slight increase in mortality risk. Nevertheless, despite the absence of a clear association between synchronicity and survival outcomes, our results also showed that patients with synchronous CRLM tend to have more variants, higher baseline CEA levels, and a higher tumor burden in terms of number and size of CRLM. Therefore, synchronicity was not significantly associated with survival benefit but may reflect tumor biology.

These findings suggest that the initially observed survival advantage of late metachronous CRLM likely reflects differences in baseline patient-, tumor-, and treatment-related characteristics, such as number and size of metastases and eligibility for local treatment, rather than the timing of detection itself. Moreover, patients with early metachronous CRLM had worse OS in the AmCORE cohort, while patients with late metachronous CRLM showed better OS in the up-front locally treated subgroup. This likely reflects that only AmCORE patients underwent up-front local treatment, whereas CAIRO5 predominantly enrolled synchronous patients treated with induction systemic therapy, thereby attenuating differences between timing groups. Although the timing of CRLM detection may serve as a surrogate for underlying tumor biology, it is not independently associated with prognosis after adjusting for other variables. Notably, the improved survival observed in patients undergoing local treatment highlights the critical role of local treatment and thereby a favorable response to systemic therapy, as conversion to local treatment largely depends on this response.

Previous studies^4,5,6,7^ have suggested that synchronous CRLM may reflect a more aggressive tumor phenotype and thus carry a worse prognosis. However, these studies often vary in definitions of CRLM timing and may not consistently adjust for relevant confounders. Our findings align with reports that did not demonstrate an independent prognostic association with detection timing after multivariable adjustment.^7^ These findings also support the ESMO guidelines, which emphasize local treatment and response to systemic therapy as key factors in the management of metastatic CRC.^15^

Methodological issues complicate interpretation of prognostic studies classifying CRLM by detection time. First, timing of CRLM is inherently influenced by the frequency and timing of imaging, especially in patients with metachronous CRLM. In clinical practice, follow-up imaging after primary tumor detection and/or resection typically occurs after a few months, limiting the opportunity to detect truly synchronous lesions. Thus, the classification of CRLM as metachronous depends on both surveillance strategy as well as tumor biology. In our study, imaging protocols were comparable between cohorts, with both the AMCORE registry and CAIRO5 trial performing follow-up imaging every 4 months in the first year. Therefore, any potential impact of differences in surveillance intensity on CRLM classification is expected to be minimal. Second, survival analyses from the time of primary tumor diagnosis introduce potential immortal time bias, as patients with early or late metachronous CRLM must survive long enough to receive such a classification.^14^ As a result, survival estimates for patients with metachronous CRLM may be artificially inflated, since early deaths are by definition excluded from this group.^14^ This bias could be minimized by calculating survival from the date of CRLM diagnosis rather than from the primary tumor diagnosis. This bias is evident in the study of Garajova et al^6^ in which OS was calculated in 2 ways: (1) from date of colon cancer diagnosis; and (2) from date of liver surgery.^1^ Results demonstrated that patients with metachronous CRLM had significantly improved OS compared with those with synchronous CRLM when calculating OS from date of colon cancer diagnosis, while the OS benefit disappeared when OS was calculated from date of first liver surgery. The present study showed similar results regarding immortal time bias, in which improved OS outcomes were found when calculating OS from diagnosis of the primary tumor compared with diagnosis of CRLM (Figure 2; eFigure 1 in Supplement 1). Third, some studies do not clearly report the starting point used for OS calculations (ie, whether from the time of primary tumor diagnosis, the diagnosis of CRLM, or initiation of liver treatment), thereby limiting the ability to interpret or compare prognostic outcomes across studies.^8,16,17^ The lack of methodological transparency hampers the validity of conclusions regarding the prognostic significance of timing of diagnosis, both in univariable and multivariable analyses.

The current results underscore that the prognostic association with the timing of CRLM detection is largely mediated by associated disease characteristics and treatment feasibility. Although patients with synchronous CRLM tend to have higher baseline CEA levels and more metastases, it remains unclear whether synchronous CRLM reflects a more aggressive tumor biology or is simply a consequence of increased tumor burden. One hypothesis could be that synchronous CRLM may reflect early systemic dissemination of disease driven by a more aggressive biological phenotype and greater metastatic potential, whereas metachronous disease reflects a more indolent course. In this context, molecular biomarkers (eg, *RAS*/*BRAF* variant status or circulating tumor DNA) and quantitative volumetric metrics (eg, total tumor volume) may allow for a more precise separation of biological aggressiveness from tumor burden.^18,19^ Clinicians should therefore prioritize individualized treatment planning based on disease burden, biological features, and local treatment options, rather than relying on the timing of CRLM detection as a primary prognostic determinant.

Future prospective studies should use uniform imaging and follow-up schedules to confirm these findings. An improved uniform diagnostic framework will help distinguish whether the timing of CRLM detection reflects true biological differences, such as disease progression rate, metastatic potential, or treatment responsiveness, or whether it is primarily a consequence of surveillance intensity.^15^ In addition, testing for MMR status, *RAS* and *BRAF* variants, should be performed in all patients at the time of metastatic CRC diagnosis, as recommended by the ESMO guidelines.^15^ This could give more insights in the biology-driven management of CRLM, and could elucidate whether synchronous CRLM could truly represent biology or simply an increased tumor burden. Last, future research should encourage ESMO guidelines and E-AHPBA consensus to consider standardized methodological quality in CRLM studies. This includes mandating survival analyses from the time of CRLM diagnosis (rather than primary tumor diagnosis) as the standard starting point to eliminate immortal time bias and to clearly define and report the starting point of survival calculations. Such a shift would substantially enhance the comparability, reliability, and validity of findings across the broader CRLM literature, addressing a pervasive issue that undermines current interpretations.

### Limitations

This study has several limitations. First, combining 2 heterogeneous cohorts (AmCORE and CAIRO5) introduces the potential for confounding by indication and selection bias related to treatment allocation. As a result, there were substantial differences in baseline disease burden, variant status availability, follow-up protocols, and treatment approach. Although this may have influenced treatment allocation and outcomes, stratified analyses by cohort and by treatment intent demonstrated consistent associations, suggesting limited effect modification across cohorts. However, this heterogeneity may also be considered a strength, as it captures a diverse cohort of patients with CRLM, offering insights into a wide array of clinical situations. Stratified analyses by treatment intent and cohort showed consistent results, mitigating the potential for effect modification by disease severity. Second, the wide inclusion period in the AmCORE cohort introduces temporal heterogeneity, as imaging quality, local treatment techniques, and systemic therapy strategies have evolved substantially over the past 25 years. These advancements likely improved CRLM detection, resectability, and treatment efficacy in later eras, potentially confounding cohort comparisons and biasing outcomes toward more favorable results. Last, molecular profiles were not consistently available, particularly in AmCORE patients that did not receive systemic therapy, limiting molecular subgroup analyses. Missingness for variant status was primarily prevalent in earlier time periods and in patients who underwent up-front local treatment; this missing-at-random pattern may have biased our findings. However, the results of separate analyses in the CAIRO5 cohort, in which variant status data were available for all patients, were globally similar to those in the AmCORE registry, indicating that the impact of missingness may have been limited in our study. A further limitation concerns the definition of early vs late metachronous disease and the potential for immortal time bias. Although we applied the E-AHPBA consensus definition for consistency with existing literature, the cutoff is inherently arbitrary and may preferentially select patients with more favorable tumor biology. More sophisticated causal inference approaches, such as clone-censor-weighting methods, could better address immortal time bias but were not applied in the present study and should be considered in future analyses. Additionally, the observational nature of the study introduces the potential for treatment-confounder feedback, whereby baseline tumor burden influences induction therapy, which in turn affects resectability and subsequent treatment decisions, potentially leading to residual confounding and biased effect estimates. More advanced causal inference approaches, such as G-methods, could better address such time-varying confounding, but require detailed longitudinal data on time-varying covariates that were not consistently available in the present dataset.

## Conclusion

In conclusion, synchronous detection of CRLM was not independently associated with survival but could instead reflect tumor biology. Although the timing of CRLM detection was associated with unadjusted survival outcomes, it was not an independent factor of OS after adjustment for tumor burden, variant status, and treatment variables. In this analysis, the ability to undergo local treatment emerged as the strongest variable associated with survival. Future research should focus on identifying markers that better reflect tumor biology and on preventing immortal time bias to enable more accurate and clinically meaningful conclusions regarding the prognostic relevance of metastatic disease onset.

## Notes

Supplement 1. eAppendix 1. Characteristics, Diagnostic Work-Up and Follow-Up of Both Cohorts eAppendix 2. Definitions of Patient-, Tumor-, and Treatment-Related Characteristics eAppendix 3. Secondary Outcomes eFigure 1. Flowchart of Included and Excluded Patients eFigure 2. Kaplan-Meier Curve for Unadjusted Overall Survival From Date of Primary Tumor Diagnosis in Patients With Synchronous, Early Metachronous and Late Metachronous CRLM eFigure 3. Kaplan-Meier Curves for Unadjusted Overall Survival From Date of Diagnosis of CRLM From the AmCORE Registry and CAIRO5 Trial in Patients With Synchronous, Early Metachronous and Late Metachronous CRLM eFigure 4. Kaplan–Meier Curves for Unadjusted Distant Progression-Free Survival in Patients With Synchronous, Early Metachronous, and Late Metachronous CRLM eTable 1. Patient-, Tumor- and Treatment-Related Characteristics of AmCORE Registry and CAIRO5 Trial eTable 2. Patient-, Tumor- and Treatment-Related Characteristics per Treatment Strategy eReferences.

Supplement 2. Data Sharing Statement
