# Supplementary material for: Time of Detection of Liver Metastases and Survival in Patients With Colorectal Cancer
Source: JAMA Netw Open. 2026 Mar 17;9(3):e262088. doi: 10.1001/jamanetworkopen.2026.2088 (PMC13446806; doi:10.1001/jamanetworkopen.2026.2088)
Supplement: Supplementary file 1 [file jamanetwopen-e262088-s001.pdf]

Supplemental Online Content

Kemna R, Schulz, HH, Ali M, et al.Time of detection of liver metastases and survival in patients with colorectal cancer. *JAMA Netw Open*. 2026;9(3):e262088. doi:10.1001/jamanetworkopen.2026.2088

eAppendix 1. Characteristics, Diagnostic Work-Up and Follow-Up of Both Cohorts

eAppendix 2. Definitions of Patient-, Tumor-, and Treatment-Related Characteristics

eAppendix 3. Secondary Outcomes

eFigure 1. Flowchart of Included and Excluded Patients

eFigure 2. Kaplan-Meier Curve for Unadjusted Overall Survival From Date of Primary Tumor Diagnosis in Patients With Synchronous, Early Metachronous and Late Metachronous CRLM

eFigure 3. Kaplan-Meier Curves for Unadjusted Overall Survival From Date of Diagnosis of CRLM From the AmCORE Registry and CAIRO5 Trial in Patients With Synchronous, Early Metachronous and Late Metachronous CRLM

eFigure 4. Kaplan–Meier Curves for Unadjusted Distant Progression-Free Survival in Patients With Synchronous, Early Metachronous, and Late Metachronous CRLM

eTable 1. Patient-, Tumor- and Treatment-Related Characteristics of AmCORE Registry and CAIRO5 Trial

eTable 2. Patient-, Tumor- and Treatment-Related Characteristics per Treatment Strategy

eReferences

This supplemental material has been provided by the authors to give readers additional information about their work.

© 2026 Kemna R et al. *JAMA Network Open*.

eAppendix 1. Characteristics, Diagnostic Work-Up and Follow-Up of Both Cohorts

AmCORE Registry

The AmCORE registry is a single-center registry at the Amsterdam University Medical Center and prospectively includes all patients that receive local treatment for CRLM, regardless of neoadjuvant or induction systemic treatment prior to local treatment. The AmCORE registry was approved by the affiliated Institutional Review Board (METc number 2021.0121). In this retrospective study, patients were included from January 2000 until December 2024 and were eligible for inclusion if they received their first local treatment for CRLM through surgical resection, thermal ablation or a combination of both. Patients were excluded in case of extrahepatic metastases at the time of CRLM diagnosis or if data were missing regarding the date of diagnosis and/or follow-up.

Adhering to national and international guidelines, all patients in this cohort underwent diagnostic tests, including cross-sectional imaging[1,2]. Contrast-enhanced computed tomography (ceCT) of the chest and abdomen was available for every patient. If considered necessary by the local MDT, an additional positron emission tomography (PET) scan was performed to detect possible distant metastases and local lymph node metastases. In case of CRLM, additional contrast-enhanced magnetic resonance imaging (ceMRI) with diffusion-weighted images (DWI) was performed to evaluate local treatment options[3,4].

The most appropriate local treatment strategy was determined by a multidisciplinary team (MDT), which included an oncological or hepatobiliary surgeon, (interventional) radiologist, medical oncologist, radiation oncologist, nuclear medicine physician, gastroenterologist, and pathologist.

During the first year after local treatment of CRLM, all patients received diagnostic ceCT of the chest and abdomen every 4 months, in accordance with national and international guidelines. Progressive disease was defined as a new solid mass on ceCT and/or ceMRI or new 18F-FDG PET-CT avidity at any location, in accordance with national and international guidelines[1,2]. During the second and third year after local treatment, ceCT of the chest and abdomen was performed every 6 months. During the fourth and fifth year after local treatment, a ceCT of the chest and abdomen was performed every year.

Phase 3 CAIRO5 trial

The CAIRO5 trial is a multicenter randomized phase 3 trial of the Dutch Colorectal Cancer Group (CAIRO5 trial number: NCT02162563). In this trial, patients with initially unresectable liver-only metastases of CRC were randomized between different systemic treatment combinations based on primary tumor site and genetic tumor mutation status (RAS/BRAFV600E).

Prior to randomization in the CAIRO5 trial, the following test were available: ceCT scan thorax and abdomen prior to randomization; a PET scan to confirm the absence of extrahepatic disease was recommended but not

© 2026 Kemna R et al. *JAMA Network Open*.

mandatory; a MRI scan of liver to assess resectability was recommended but not mandatory. Prior to each systemic treatment cycle until end of treatment or progression, physical examination and laboratory test were carried out. Every 4 cycles (8 weeks) until progression, patients received a ceCT scan abdomen and thorax. Resectability or unresectability of CRLM was assessed centrally by an expert panel of liver surgeons and radiologists, at baseline and every 2 months thereafter by predefined criteria. If patients had undergone resection of liver metastases, follow-up was carried out according to the national guideline: ultrasound or ceCT scan of the liver every 6 months for 2 years, then every 12 months up to 5 years after surgery. Assessment of serum CEA was performed every 3-6 months for 3 years, then every 5 months up to 5 years after surgery[5].

© 2026 Kemna R et al. *JAMA Network Open*.

eAppendix 2. Definitions of Patient-, Tumor-, and Treatment-Related Characteristics

Age was reported as age at time of diagnosis of CRLM in the AmCORE registry, and determined at randomization in the CAIRO5 trial. The primary tumor location was subdivided in rectum (rectosigmoid and rectum); right colon (caecum up to and including transverse colon); and left colon (splenic flexure up to and including sigmoid). The Fong score was calculated by the predefined criteria of the Fong Clinical Risk Score for Colorectal Cancer Recurrence, consisting of five factors: node-positive primary tumor; disease-free interval <12 months (from diagnosis of primary tumor to discovery of liver metastasis); >1 tumor; preoperative CEA level >200ug/mL; size of largest tumor >5cm. Each of the five factors is assigned one point, with a total possible score ranging from 0 to 5. The different types of local treatment included surgery, thermal ablation, a combination of both, or no local treatment. Systemic treatment was categorized into no systemic treatment, induction systemic therapy followed by local treatment, and induction systemic therapy not followed by local treatment.

© 2026 Kemna R et al. *JAMA Network Open*.

eAppendix 3. Secondary Outcomes

Secondary outcomes included OS from diagnosis of primary tumor in both cohorts, and distant progression-free survival in the AmCORE cohort (**Supplement S3**).

First, OS from diagnosis of primary tumor was assessed for both cohorts (AmCORE and CAIRO5), defined as time from diagnosis of the primary tumor to death from any cause, based on the timing of CRLM diagnosis, i.e., synchronous, early and late metachronous. Second, for the AmCORE cohort, distant progression-free survival (DPFS) was assessed, defined as the time from local treatment of CRLM to the development of distant disease progression, death from any cause and any factor influencing the decision not to treat disease progression was considered a competing risk. DPFS was assessed in two ways. First, comparisons were made among all included patients based on the timing of CRLM diagnosis: synchronous, early and late metachronous. Second, DPFS was compared among patients who received upfront local treatment, and those who received induction systemic therapy followed by local treatment. According to the SIO- and DATECAN-initiated consensus guidelines, distant recurrence includes both new CRLM at different sites and new extrahepatic metastases[6].

© 2026 Kemna R et al. *JAMA Network Open*.

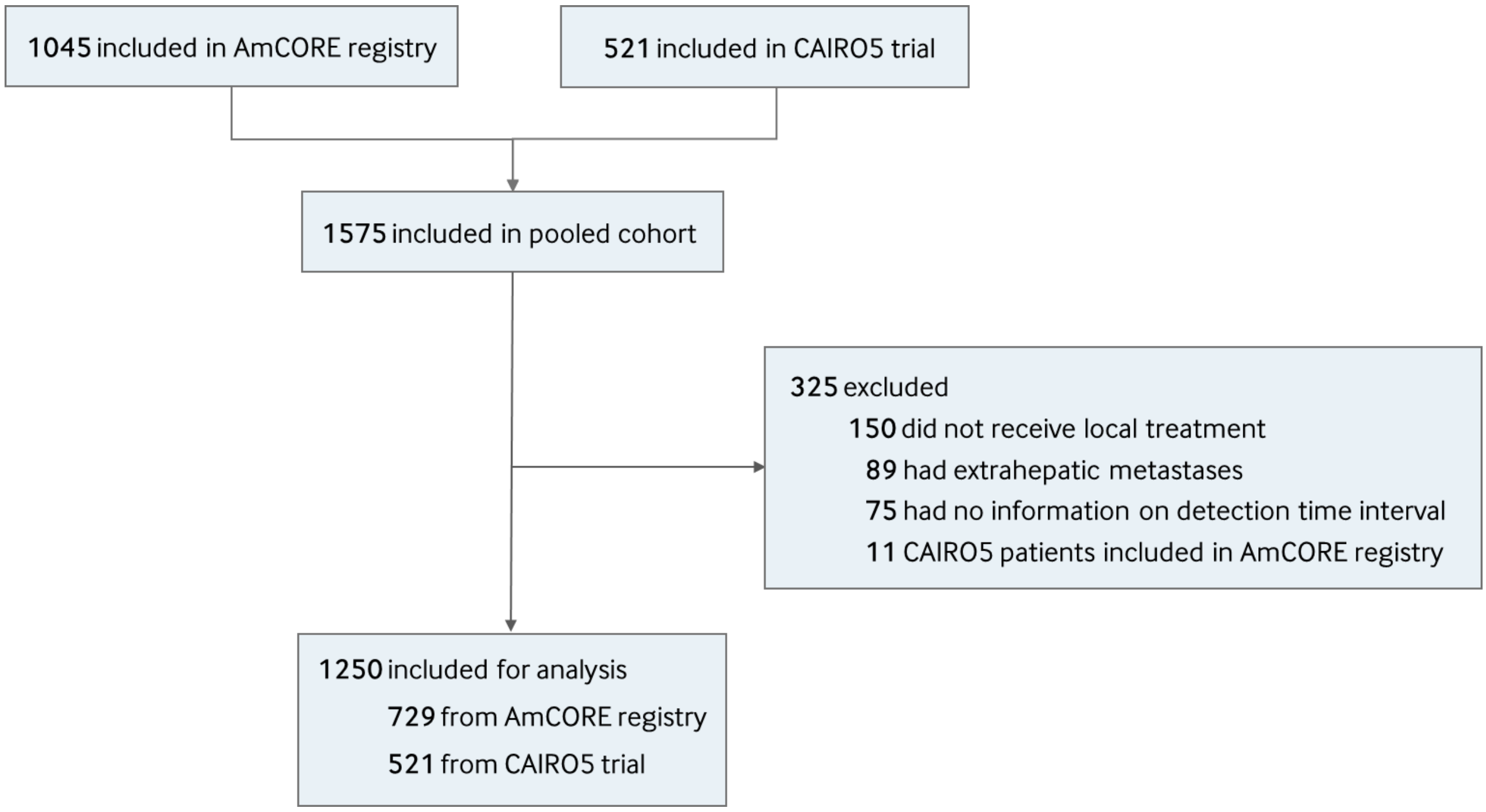

eFigure 1. Flowchart of Included and Excluded Patients

A total of 325 patients were excluded, all originating from the AmCORE registry. Specifically, 150 patients did not receive local treatment, and therefore no follow-up data were available. In addition, 89 patients presented with extrahepatic metastases, and 75 patients lacked information on the detection time interval. Lastly, 11 CAIRO5 patients were also registered in the AmCORE database and were excluded to prevent duplicate inclusion.

© 2026 Kemna R et al. *JAMA Network Open*.

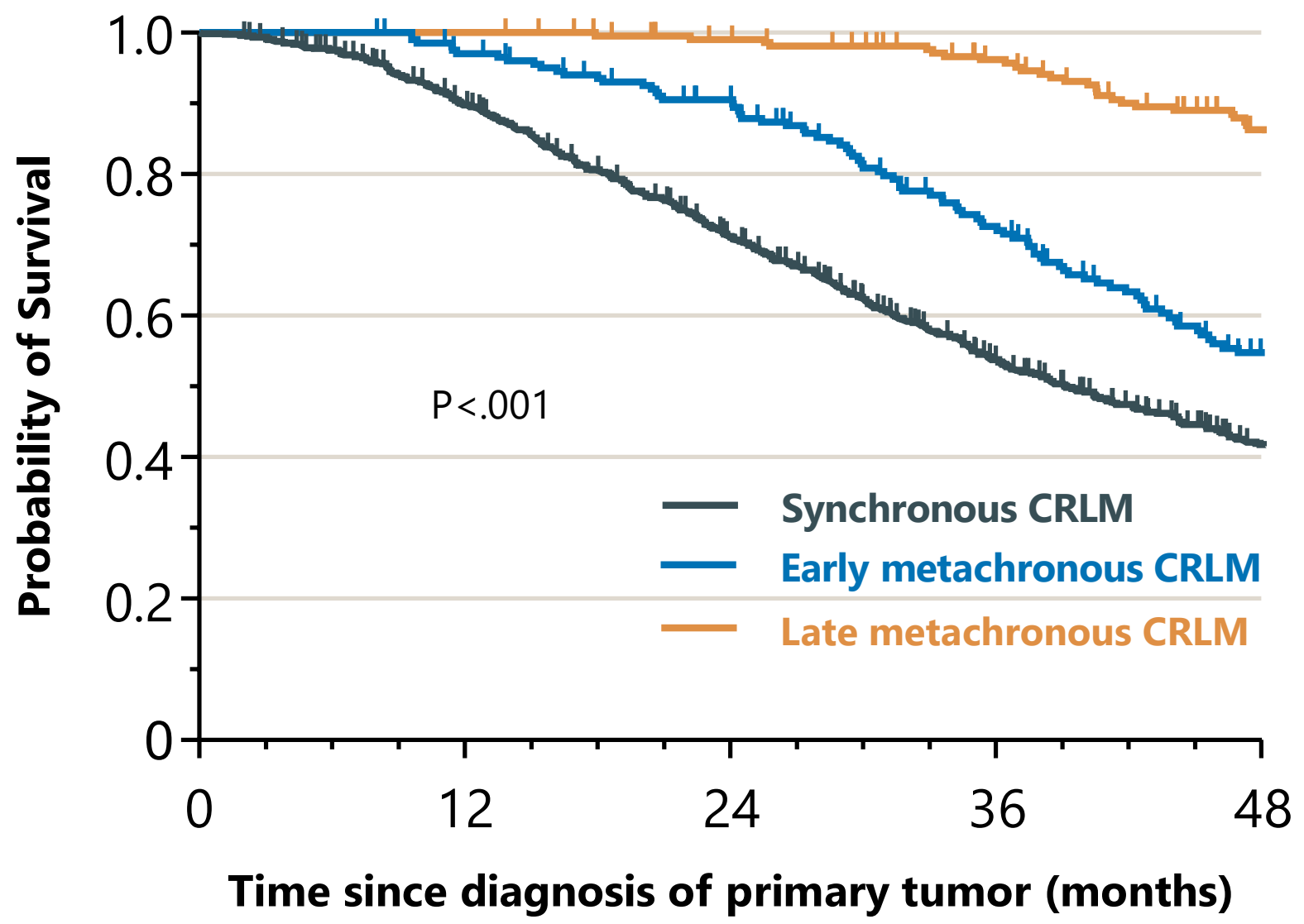

No. at risk

	807 (0)	693 (80)	514 (219)	337 (338)	215 (407)
	207 (0)	197 (6)	175 (19)	131 (52)	82 (82)
	222 (0)	222 (0)	211 (2)	192 (8)	159 (27)

eFigure 2. Kaplan-Meier Curve for Unadjusted Overall Survival From Date of Primary Tumor Diagnosis in Patients With Synchronous, Early Metachronous and Late Metachronous CRLM

Compared with log-rank test (P <.001). Dark blue indicates patients with synchronous CRLM, light blue early metachronous CRLM and orange metachronous CRLM. Unadjusted OS Kaplan-Meier curves from diagnosis of the primary tumor. The unadjusted HR for OS in patients with early metachronous CRLM compared to synchronous CRLM was 0,64 (95% CI 0.52 to 0.79, P <.001). Patients with late metachronous CRLM had an unadjusted HR of 0.28 (95% CI 0.22 to 0.36, P <.001). In patients with synchronous CRLM, the median OS was 39.2 months (IQR 36.1 to 44.0 months) with 1-, 3-, and 5-year OS rates of 89.8% (95% CI 87.7% to 92.0%), 53.8% (95% CI 50.2% to 57.6%), and 31.4% (95% CI 27.7% to 35.5%), respectively. The patients with early metachronous CRLM showed a median OS of 54.2 months (IQR 45.6 to 65.9 months) with 1-, 3-, and 5-year OS rates of 97.1% (95% CI 94.8% to 99.4%), 72.6% (95% CI 66.5% to 79.3%), and 46.9% (95% CI 39.8% to 55.2%), respectively, versus a median OS of 97.2 months (IQR 87.3 to v153.3 months) with a 1-, 3-, and 5-year OS rates of 100% (95% CI 100% to 100%), 96.2% (95% CI 93.6% to 98.8%), and 77.7% (95% CI 27.0% to 83.9%), in patients with late metachronous CRLM.

© 2026 Kemna R et al. *JAMA Network Open*.

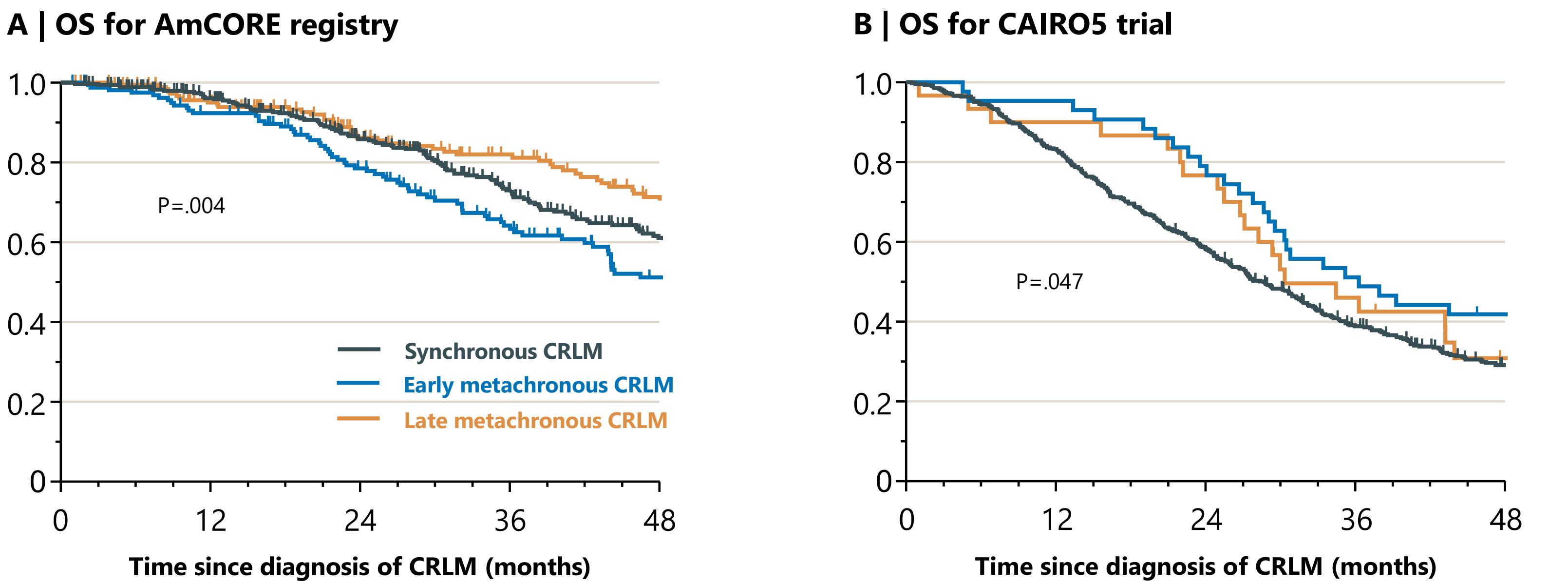

No. at risk (A | OS for AmCORE registry)

	0	12	24	36	48
Synchronous CRLM	360 (0)	312 (13)	242 (44)	169 (77)	111 (102)
Early metachronous CRLM	164 (0)	143 (12)	111 (32)	79 (51)	50 (65)
Late metachronous CRLM	192 (0)	167 (9)	129 (23)	107 (29)	81 (42)

No. at risk (B | OS for CAIRO5 trial)

	0	12	24	36	48
Synchronous CRLM	448 (0)	373 (75)	261 (186)	157 (270)	101 (307)
Early metachronous CRLM	43 (0)	41 (2)	34 (9)	22 (21)	17 (25)
Late metachronous CRLM	30 (0)	27 (3)	23 (7)	13 (16)	7 (20)

eFigure 3. Kaplan-Meier Curves for Unadjusted Overall Survival From Date of Diagnosis of CRLM From the AmCORE Registry and CAIRO5 Trial in Patients With Synchronous, Early Metachronous and Late Metachronous CRLM

Compared with log-rank test (AmCORE: P =.004 and CAIRO5: P = .047). Dark blue indicates patients with synchronous CRLM, light blue early metachronous CRLM and orange metachronous CRLM.

© 2026 Kemna R et al. *JAMA Network Open*.

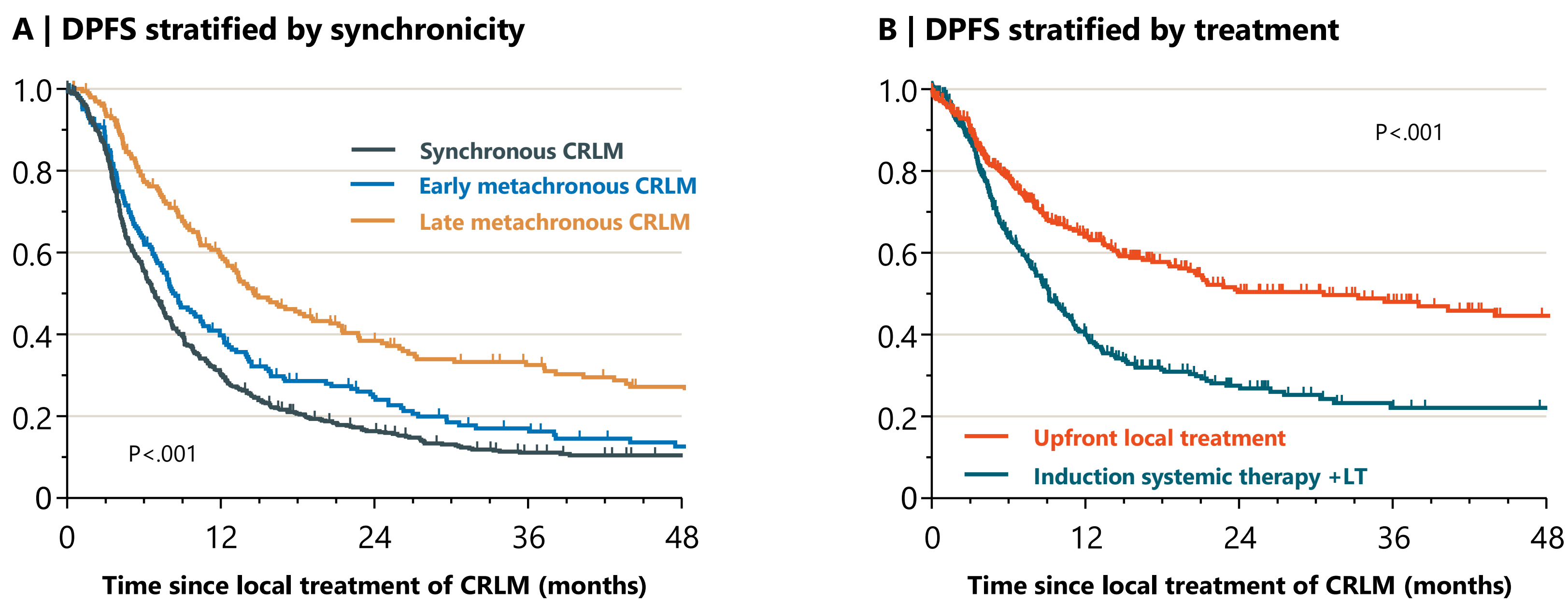

No. at risk (A)

	0	12	24	36	48
Synchronous CRLM	566 (0)	159 (377)	75 (446)	39 (468)	29 (470)
Early metachronous CRLM	186 (0)	69 (107)	37 (132)	22 (143)	13 (148)
Late metachronous CRLM	203 (0)	107 (78)	62 (114)	44 (123)	32 (130)

No. at risk (B)

	0	12	24	36	48
Upfront local treatment	425 (0)	181 (213)	98 (276)	67 (291)	42 (301)
Induction systemic therapy +LT	484 (0)	124 (336)	52 (397)	26 (416)	21 (419)

eFigure 4. Kaplan–Meier Curves for Unadjusted Distant Progression-Free Survival in Patients With Synchronous, Early Metachronous, and Late Metachronous CRLM

Compared with log-rank test (P < .001). Dark blue indicates patients with synchronous CRLM, light blue early metachronous CRLM and orange with metachronous CRLM; and **B) upfront locally treatable CLRM, compared to patients that received induction systemic therapy prior to local treatment**. Compared with log-rank test (P < .001). Red indicates patients with upfront locally treatable CLRM, and blue patients that received induction systemic therapy prior to local treatment. In the synchronous CRLM group, the 1-, 3-,

© 2026 Kemna R et al. *JAMA Network Open*.

and 5-year DPFS rates were 30.3% (95% CI 26.7 to 34.5), 11.1% (95% CI 8.6 to 14.3), and 9.3% (95% CI 6.9 to 12.6), respectively. Patients with early metachronous CRLM had corresponding rates of 39.8% (95% CI 33.2 to 47.7), 17.1% (95% CI 12.1 to 24.1), and 10.4% (95% CI 6.3 to 17.4). In the late metachronous CRLM group, the 1-, 3-, and 5-year DPFS rates were 59.0% (95% CI 52.4 to 66.5), 32.6% (95% CI 26.3 to 40.4), and 19.7% (95% CI 14.0 to 27.6), respectively.

© 2026 Kemna R et al. *JAMA Network Open*.

eTable 1. Patient-, Tumor- and Treatment-Related Characteristics of AmCORE Registry and CAIRO5 Trial

		AmCORE<br>N = 729	CAIRO5<br>N = 521	P-value
**Patient-related characteristics**				
**Sex**	Male	499 (68.6)	324 (62.2)	.02
	Female	228 (31.4)	197 (37.8)	
**Age (years)**	Median (IQR)	(65 (57 to 73)	62 (54 to 69)	<.001
**Tumor-related characteristics**				
**Detection time interval of CRLM**	Synchronous	369 (50.6)	448 (86.0)	<.001
	Early metachronous	165 (22.6)	43 (8.3)	
	Late metachronous	195 (26.8)	30 (5.8)	
**Primary tumor location**	Rectum	264 (36.3)	191 (36.7)	.43
	Right-sided colon	166 (22.8)	133 (25.5)	
	Left-sided colon	298 (40.9)	197 (37.8)	
**Molecular profile**	RAS wildtype	125 (17.2)	271 (52.0)	<.001
	mutation	88 (12.1)	250 (48.0)	
	unknown	514 (70.7)	0 (0)	
	BRAF wildtype	183 (25.1)	491 (94.2)	<.001
	mutation	15 (2.1)	30 (5.8)	
	unknown	530 (72.8)	0 (0)	
	MMR proficient	318 (43.7)	83 (15.9)	.005
	deficient	7 (1.o)	(0.4)	
	unknown	403 (55.4)	(83.7)	

© 2026 Kemna R et al. *JAMA Network Open*.

**Number CRLM at diagnosis**	Median (IQR)	2 (1 to 4)	12 (7 to 22)	<.001
**Largest tumor diameter at diagnosis (mm)**	Median (IQR)	24 (17 to 35)	41 (27 to 66)	<.001
**CEA levels at diagnosis**	Median (IQR)	7 (4 to 22)	43 (10 to 251)	<.001
**Fong score**	Fong 1	120 (50.0)	12 (2.3)	.005
	Fong 2	79 (32.9)	196 (37.6)	
	Fong 3	36 (15.0)	194 (37.2)	
	Fong 4	5 (2.1)	109 (20.9)	
	Fong 5	0 (0)	10 (1.9)	
**Treatment-related characteristics**				
**Local treatment**	Resection	262 (35.9)	150 (28.8)	<.001
	Thermal ablation (TA)	256 (35.1)	18 (3.5)	
	Resection and TA	211 (28.9)	144 (27.6)	
	No local treatment	0 (0)	209 (40.1)	
**Systemic treatment (induction)**	No	449 (66.7)	0 (0)	<.001
	Induction systemic therapy *followed by* local treatment	224 (33.3)	312 (59.9)	
	Induction systemic therapy *not followed by* local treatment	0 (0)	209 (40.1)	

Categorical variables are reported as number of patients (%), continuous variables are reported as median (IQR1 to IQR3).
Abbreviations: RAS = Rat Sarcoma Viral Oncogene Homolog; BRAF = V-raf Murine Sarcoma Viral Oncogene Homolog B; wt = wildtype; mut = mutation; MSS = Microsatellite Stability; MSI = Microsatellite Instability; CEA = carcinoembryonic antigen; TA= thermal ablation; this includes microwave ablation (MWA) and radiofrequency ablation (RFA)induction or neoadjuvant systemic treatment before local treatment of CRLM.

© 2026 Kemna R et al. *JAMA Network Open*.

eTable 2. Patient-, Tumor- and Treatment-Related Characteristics per Treatment Strategy

		Total<br>N = 1194	Upfront local treatment<br>N = 449	Induction systemic therapy followed by local treatment<br>N = 536	Induction systemic therapy not followed by local treatment<br>N = 209	P-value
**Patient-related characteristics**						
**Patient cohort**	AmCORE registry	729 (56.4)	499 (100)	224 (41.8)	0 (0)	<.001
	CAIRO5 trial	521 (43.6)	0 (0)	312 (58.2)	209 (100)	
**Sex**	Male	823 (65.3)	307 (68.4)	345 (64.5)	127 (60.8)	.14
	Female	425 (34.7)	142 (31.6)	190 (35.5)	82 (39.2)	
**Age (years)**	Median (IQR)	63 (56-72)	(67 (59-75)	61 (54-70)	63 (55-71)	<.001
**Tumor-related characteristics**						
**Detection time interval of CRLM**	Synchronous	817 (66.2)	187 (41.6)	411 (76.7)	193 (92.3)	<.001
	Early metachronous	208 (16.2)	115 (25.6)	71 (13.2)	8 (3.8)	
	Late metachronous	225 (17.5)	147 (32.7)	54 (10.1)	8 (3.8)	
**Primary tumor location**	Rectum	455 (36.1)	160 (35.6)	197 (36.8)	74 (35.4)	.003
	Right-sided colon	299 (24.0)	113 (25.2)	233 (19.8)	67 (32.5)	
	Left-sided colon	495 (39.9)	176 (39.2)	106 (43.5)	68 (32.1)	
**Molecular profile**	RAS wildtype	396 (32.8)	52 (11.6)	245 (45.7)	94 (45.0)	<.001
	mutation	338 (28.1)	50 (11.2)	170 (31.7)	115 (55.0)	
	unknown	514 (39.1)	345 (77.2)	121 (22.6)	0 (0)	
	BRAF wildtype	674 (55.9)	93 (20.8)	385 (71.8)	189 (90.4)	<.001
	mutation	45 (3.8)	4 (0.9)	21 (3.9)	20 (9.6)	

© 2026 Kemna R et al. *JAMA Network Open*.

	unknown	530 (40.3)	351 (78.3)	130 (24.3)	0 (0)	
	MMR proficient	401 (32.9)	186 (41.5)	206 (38.4)	0 (0)	<.001
	deficient	9 (0.7)	4 (0.9)	4 (0.7)	0 (0)	
	unknown	839 (66.5)	258 (57.6)	326 (60.8)	209 (100)	
**Number CRLM at diagnosis**	Median (IQR)	5 (2-12)	2 (1-3)	8 (4-12)	20 (11-34)	<.001
**Largest tumor diameter at diagnosis (mm)**	Median (IQR)	30 (20-49)	23 (16-31)	33 (22-53)	50 (34-72)	<.001
**CEA levels at diagnosis**	Median (IQR)	15.0 (5.0-95.0)	5.6 (3.0-15.0)	20.6 (6.8-102.8)	98.6 (14.2-366.0)	<.001
**Fong score**	Fong 1	132 (17.0)	105 (57.7)	18 (5.1)	3 (1.4)	<.001
	Fong 2	275 (35.9)	56 (30.8)	153 (43.7)	57 (27.3)	
	Fong 3	230 (30.4)	19 (10.4)	118 (33.7)	88 (42.1)	
	Fong 4	114 (15.4)	2 (1.1)	54 (15.4)	58 (27.8)	
	Fong 5	10 (1.3)	0 (0)	7 (2.0)	3 (1.4)	
**Treatment-related characteristics**						
**Local treatment**	Resection	412 (31.2)	177 (39.4)	196 (36.6)	0 (0)	<.001
	Thermal ablation (TA)	274 (22.8)	177 (39.4)	95 (17.7)	0 (0)	
	Resection and TA	355 (28.5)	95 (21.2)	245 (45.7)	0 (0)	
	No local treatment	209 (17.5)	0 (0)	0 (0)	209 (100)	

Categorical variables are reported as number of patients (%), continuous variables are reported as median (IQR1 to IQR3).
Abbreviations: RAS = Rat Sarcoma Viral Oncogene Homolog; BRAF = V-raf Murine Sarcoma Viral Oncogene Homolog B; wt = wildtype; mut = mutation; MSS = Microsatellite Stability; MSI = Microsatellite Instability; CEA = carcinoembryonic antigen; TA= thermal ablation; this includes microwave ablation (MWA) and radiofrequency ablation (RFA)induction or neoadjuvant systemic treatment before local treatment of CRLM.

© 2026 Kemna R et al. *JAMA Network Open*.

Information regarding systemic therapy prior to local treatment was missing for 56 patients. Among 1185 patients, 440 (37.1%) received upfront local treatment, 536 (45.2%) received induction systemic therapy followed by local treatment, and 209 (17.6%) received induction systemic therapy without subsequent local treatment. Conversely, the proportion receiving no systemic therapy prior to local treatment was greater in the early and late metachronous groups than in synchronous CRLM (59.3% and 70.3% vs 23.6%; P<.001), this upfront local treatment group consisted solely of patients from the AmCORE cohort. Within the systemic-therapy cohorts, molecular profiling (RAS, BRAF, and MMR) was more frequently available than in the upfront locally treatable group, where these statuses were often unknown. Regarding local treatment modalities, combined resection plus thermal ablation procedures was more common among patients who received induction systemic therapy followed by local treatment, whereas in the upfront locally treatable group resection and thermal ablation were used in roughly equal proportions

© 2026 Kemna R et al. *JAMA Network Open*.

eReferences

1. Schulz HH, Dijkstra M, van der Lei S, et al. Local Treatment of Colorectal Liver Metastases in the Presence of Extrahepatic Disease: Survival Outcomes from the Amsterdam Colorectal Liver Met Registry (AmCORE). *Cancers (Basel)*. Mar 8 2024;16(6)doi:10.3390/cancers16061098

2. Cervantes A, Adam R, Roselló S, et al. Metastatic colorectal cancer: ESMO Clinical Practice Guideline for diagnosis, treatment and follow-up. *Ann Oncol*. Jan 2023;34(1):10–32. doi:10.1016/j.annonc.2022.10.003

3. Görgec B, Hansen I, Kemmerich G, et al. Clinical added value of MRI to CT in patients scheduled for local therapy of colorectal liver metastases (CAMINO): study protocol for an international multicentre prospective diagnostic accuracy study. *BMC Cancer*. Oct 18 2021;21(1):1116. doi:10.1186/s12885-021-08833-1

4. Görgec B, Hansen IS, Kemmerich G, et al. MRI in addition to CT in patients scheduled for local therapy of colorectal liver metastases (CAMINO): an international, multicentre, prospective, diagnostic accuracy trial. *Lancet Oncol*. Jan 2024;25(1):137–146. doi:10.1016/s1470-2045(23)00572-7

5. Bond MJG, Bolhuis K, Loosveld OJL, et al. First-line systemic treatment strategies in patients with initially unresectable colorectal cancer liver metastases (CAIRO5): an open-label, multicentre, randomised, controlled, phase 3 study from the Dutch Colorectal Cancer Group. *Lancet Oncol*. Jul 2023;24(7):757–771. doi:10.1016/s1470-2045(23)00219-x

6. Puijk RS, Ahmed M, Adam A, et al. Consensus Guidelines for the Definition of Time-to-Event End Points in Image-guided Tumor Ablation: Results of the SIO and DATECAN Initiative. *Radiology*. Dec 2021;301(3):533–540. doi:10.1148/radiol.2021203715

© 2026 Kemna R et al. *JAMA Network Open*.
