# Supplementary material for: Time of Detection of Liver Metastases and Survival in Patients With Colorectal Cancer
Source: JAMA Netw Open. 2026 Mar 17;9(3):e262088. doi: 10.1001/jamanetworkopen.2026.2088 (PMC13446806; doi:10.1001/jamanetworkopen.2026.2088)
Supplement: Supplementary file 2 [file jamanetwopen-e262088-s002.pdf]

Data Sharing Statement

Kemna. Time of Detection of Liver Metastases and Survival in Patients With Colorectal Cancer. *JAMA Netw Open*. Published March 17, 2026. doi:10.1001/jamanetworkopen.2026.2088

Data
Data available: No

Additional Information
Explanation for why data not available: The data generated in this study are not publicly available due to information that could compromise patients' privacy and consent, but are available upon reasonable request from the corresponding author. Requests need to be accompanied by a study protocol and statistical analysis plan.
